# Summer Complaint

**Published:** 1873-07

**Authors:** 


### SUMMER COMPLAINT.

THIS means a diarrhœa, a looseness of the bowels of infants and young children, which prevails to such an extent in midsummer, especially in large cities, that the number of deaths is doubled. This is not necessarily the result of warm weather; in reality there ought to be fewer deaths among children in warm weather than in cold, because they can be more out of doors. The universal cause of "summer complaint" is injudicious feeding, and this not so much as to quality or quantity as in frequency. Children are not apt to over-eat, no more so than the brute creation, if they are fed at regular times. If mothers will only take pains to feed their little ones at regular times and at proper intervals—if this were done for one single summer—there would be thousands of little ones saved.

If a child is fed—if it is allowed to eat as much as it wants—a certain time is required for this food to be digested, dissolved, and passed out of the stomach, and then it begins to get hungry again, the hunger gradually increasing until it becomes painful if not satisfied. In grown persons it requires five hours for a regular meal to be fully digested, hence the regular meals of a family should not be less than five hours apart, during which interval not an atom of food should be eaten, ordinarily, because it has been found by ocular observation that if, after a regular meal (an hour or more) something else is eaten, the process of digestion of what was previously eaten is arrested until what is eaten later has been put into the condition of what was eaten before—precisely as when a lump of ice is put into a vessel of boiling water it ceases to boil until the ice has been brought to the boiling point. In this way a vessel of water may be kept over a hot fire all day and never come to a boil. So, if a person eats at too short intervals during any day, what was eaten at breakfast will be undigested at night. But food in the stomach is at a heat of about a hundred degrees, and if it remains so longer than five or six hours it begins to "rot," that is, to decompose, to decay, to ferment, to sour, to generate gas or wind—just as a barrel of new cider stopped up and left in a warm place begins to "sour," generate gas, and drives out the bung, or is bursted asunder, as certainly as a boiler is burst when there is no outlet to the steam. One effect of this decomposition of food is the production of "wind on the stomach," colic; this gives pain, the child cries, the mother thinks it is because it is hungry and gives it more food, thus adding fuel to the flame. The colic increases, something is given to quiet the child—that is paregoric or laudanum, in the shape of "soothing syrup"—this works like a charm, the mother is perfectly delighted, the baby goes to sleep, sleeps a long time, long enough to let nature work off the food, and long enough to let the little one get hungry again, and the same process is repeated, until at length, sometimes in a few days, nature loses the power of working off this undigested mass, because the child is weaker, as rotting food gives no nourishment.

In addition, this decaying food being "sour," is in an unnatural state; instead of passing along the intestines as a bland and strength-giving substance, it is acrid, irritating, inflames the surface of the bowels, which, like an inflamed eye, throws out water in unusual quantities, and this water, mixed with undigested particles of food, constitutes diarrhœa, cholera infantum or summer complaint, all of which mean the same thing. But as this diarrhœa is evidently weakening the child, the mother becomes uneasy and thinks it ought to be stopped; to do this the soothing syrup is resorted to; it acts like a charm; as before, the diarrhœa is stopped and the mother is delighted. She would give her last dress, if necessary, to purchase a bottle of this incomparable medicine; but in a few days convulsions come on and the child dies, or pines away, more dead than alive, for weary weeks.

Nurses often find out the "virtues" of the soothing syrup to give to fitful and restless children, and procure supplies at their own expense, to be used whenever occasion requires. One ounce of soothing syrup—that is two tablespoonfuls—contains one grain of morphine; hence, a dose for an infant three months old contains what is equal to ten drops of laudanum, which is advised to be taken every two hours; hence, in the course of a day or night, an infant takes sixty drops, or one teaspoonful of laudanum, which an intelligent grown person would be loth to swallow at one time. At a reasonable calculation the little children of this country take a hundred thousand gallons of this ruinous medicine every year.

Up to a month old a baby should be fed every two hours, increasing the distance of time by half an hour each week until it is fed five times in twenty-four hours; when it gets six months old, four times in twenty-four hours; at the end of three years, three times in twenty-four hours, which may be well continued for the remainder of life—meaning, in all these cases, that not an atom of food, whether solid or in the shape of gruel, soup, milk or sweetmeats, should be taken between. As early as possible explain the reason for this arrangement, and in the very next generation the habit would become almost universal. It is this eating between meals, of forever "nibbling," which makes three fourths of our daughters dyspeptics before they are of age. It is impossible that any intelligent mother should follow up such a course fairly, for one week, with herself or her babe, without being struck with admiration of its wholesome effects.

If babes get thirsty between the times of taking food let them have all the cool, fresh water they want.

The Board of Health for New York have recommended the following as the summer's diet for infants, when mothers cannot nurse them:

If under six months old, boil a teaspoonful of powdered barley (grind it on the coffee grinder) and a gill of water with salt, for fifteen minutes. Strain it and mix it with half as much boiled milk and a piece of loaf sugar. Give it, lukewarm, through a nursing bottle.

Keep the bottle and mouthpiece in water when not in use.

Give babies of five or six months half barley water and half boiled milk, with salt and loaf sugar.

Give older babies more milk in proportion.

When babies are very costive take oatmeal instead of barley, but be sure to cook and strain it.

When your breast milk is half enough for the infant alternate with breast and food.

In hot summer weather dip a small piece of litmus paper (obtained at a drug store) into the food before feeding; if it turns red the food is sour. If the blue paper turns red add a pinch of baking soda to the food.

Babies of six months may have beef tea or beef soup once a day, by itself or mixed with other food.

Babies of ten or twelve months may have a crust of bread and a piece of rare beefsteak to suck.

No child under two years ought to eat from your table.

The summer complaint comes from over feeding and hot and foul air Keep doors and windows open. Wash your children with cold water at least twice a day. Ten times is not too many in the hot season.

When babies throw off and purge give them nothing to eat for four to six hours, but all the cold air you can.

